# Supplementary material for: RAD51 and RAD51B Play Diverse Roles in the Repair of DNA Double Strand Breaks in Physcomitrium patens
Source: Genes (Basel). 2023 Jan 24;14(2):305. doi: 10.3390/genes14020305 (PMC9956106; doi:10.3390/genes14020305)
Supplement: Supplementary file 1 [file genes-14-00305-s001.zip › genes-2135336-supplementary.pdf]

Figure S1. Phylogenetic tree of RAD51 superfamily.

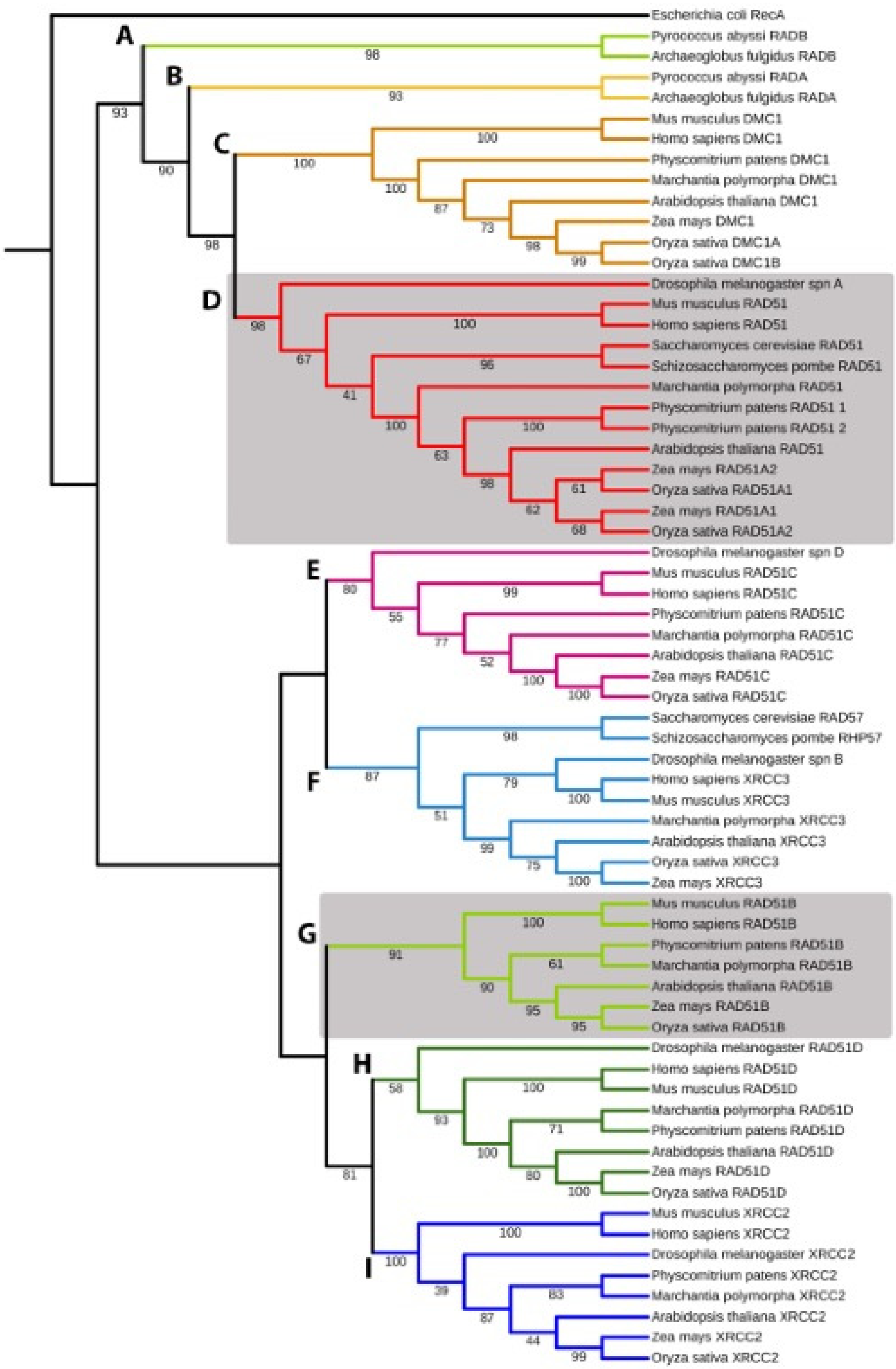

Phylogenetic tree of RAD51 superfamily inferred using maximum likelihood analysis. RecA *E. coli* was used as outgroup. The bootstrap support values are shown on the branch above. The ML log likelihood -34879.737423. The analysis included 65 amino acid sequences and 1,023 positions in the final data set. A. RADB, B. RADA, C. DCM1, D. RAD51 1 and 2, E. RAD51C, F. XRCC3, G. RAD51B, H. RAD51D and I. XRCC2. Grey boxes depicted RAD51 1 and 2 and RAD51B. Accession numbers are listed in Suppl. Table S1.

Table S1. Accession numbers of proteins used for construction of phylogenetic tree of RAD51 superfamily.

Organism	Gene name	Accession number	Database
Archaeoglobus fulgidus	RADA	1484216	NCBI
	RADB	1485325	NCBI
Pyrococcus abyssi	RADA	AJ248283.1	NCBI
	RADB	1495005	NCBI
Arabidopsis thaliana	RAD51	AT5G20850	TAIR
	RAD51B	AT2G28560	TAIR
	RAD51C	AT2G45280	TAIR
	RAD51D	AT1G07745.1	TAIR
	XRCC2	AT5G64520	TAIR
	XRCC3	AT5G57450	TAIR
	DMC1	AT3G22880.1	TAIR
Marchantia polymorpha	RAD51	Mapoly0024s0069	Phytozome
	RAD51B	Mapoly0007s0061	Phytozome
	RAD51C	Mapoly0114s0015.1	Phytozome
	RAD51D	Mapoly0014s0028.1	Phytozome
	XRCC2	Mapoly0070s0006.1	Phytozome
	XRCC3	Mapoly0009s0225.1	Phytozome
	DMC1	Mapoly0110s0049.1	Phytozome
Physcomitrium patens	RAD51_1	Pp3c11_22000V3.1	Phytozome
	RAD51_2	Pp3c7_7920V3.1	Phytozome
	RAD51B	Pp3c25_15290V3.1	Phytozome
	RAD51C	Pp3c5_12400V3.2	Phytozome
	RAD51D	Pp3c23_10930V3.2	Phytozome
	XRCC2	Pp3c1_6220V3.1	Phytozome
	DMC1	Pp3c20_7190V3.1	Phytozome
Oryza sativa	RAD51A1	LOC_Os11g40150.1	Phytozome
	RAD51A2	LOC_Os12g31370.1	Phytozome
	RAD51B	LOC_Os05g03050.1	Phytozome
	RAD51C	LOC_Os01g39630.1	Phytozome
	RAD51D	LOC_Os09g01680.1	Phytozome
	XRCC2	LOC_Os01g64990.2	Phytozome
	XRCC3	LOC_Os02g35450.1	Phytozome
	DMC1A	LOC_Os12g04980.1	Phytozome
	DMC1B	LOC_Os11g04954.1	Phytozome
Zea Mays	RAD51A1	Zm00001d041757_T001	Phytozome
	RAD1A2	Zm00001d021898_T001	Phytozome
	RAD51B	Zm00001d010986_T001	Phytozome
	Rad51C	Zm00001d044278_T001	Phytozome
	Rad51D	Zm00001d022332_T001	Phytozome
	XRCC2	Zm00001d042691_T002	Phytozome
	XRCC3	Zm00001d016839_T001	Phytozome

	DMC1	ZmPHJ40.03G333900.1	Phytozome
Homo sapiens	RAD51	Q06609	Uniprot
	RAD51B	O15315	Uniprot
	RAD51C	O43502	Uniprot
	RAD51D	O75771	Uniprot
	XRCC2	O43543	Uniprot
	XRCC3	O43542	Uniprot
	DMC1	Q14565-1	Uniprot
Mus musculus	RAD51	NP_035364.1	NCBI
	RAD51B	NP_001239491.1	NCBI
	RAD51C	NP_001278369.1	NCBI
	RAD51D	NP_001264867.1	NCBI
	XRCC2	NP_065595.2	NCBI
	XRCC3	NP_083151.1	NCBI
	DMC1	NP_034189.1	NCBI
Drosophila melanogaster	Spn-A	NP_524583.1	NCBI
	Spn-D	NP_733200	NCBI
	RAD51D	NP_610466	NCBI
	XRCC2	NP_573302.1	NCBI
	Spn-B	NP_476740.1	NCBI
S. cerevisiae	RAD51	NP_011021.3	NCBI
	RAD57	NP_010287.1	NCBI
S. pombe	RAD51	CAB90141.1	NCBI
	RHP57	NP_593627.1	NCBI

NCBI (National Center for Biotechnology Information) https://www.ncbi.nlm.nih.gov/; TAIR (The Arabidopsis Information Resource) https://www.arabidopsis.org/; UniProt https://www.uniprot.org/; Phytozome (JGI Phytozome 13) https://phytozome-next.jgi.doe.gov/

Table S2. List of rDNA qPCR primers

Target	Primer	Orientation	Sequence
qPCR 18S rDNA	Pp18S A	F	5′ CCTCTAAGAAGTTGGCCGCA 3′
	Pp18S B	R	5′ GGCCGTTCTTAGTTGGTGGA 3′
qPCR 5S rDNA	Pp5S F	F	5′ TACCAAGGCTACTACACCAGATC 3′
	Pp5S R	R	5′ AGGTCACCCATCCCAGTACTA 3′
qPCR ubiquitin	PubqFw	F	5′ ACTACCCTGAAGTTGTATAGTTCGG 3′
	PubqRev	R	5′ CAAGTCACATTACTTCGCTGTCTA 3′

Figure S2. WT, *Pprad51B* and *Pprad51-1-2* response to MMS treatment.

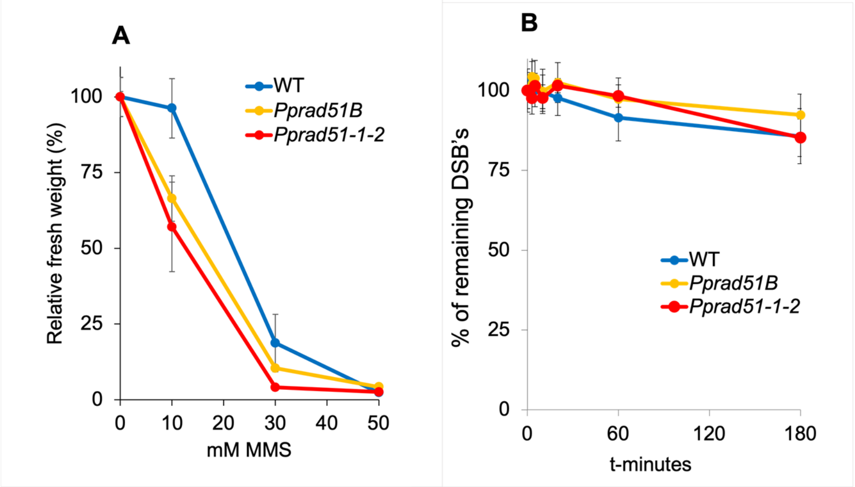

(**A)** Growth response to MMS. Explants of 7d protonemata were treated for 1 hr with 10, 30 and 50 mM MMS. After the treatment, explants were inoculated on Petri plates with drug-free BCDAT medium and weighted after 3 weeks of growth (see legend for Figure 3 in main text). WT and both lines *Pprad51B* and *Pprad51-1*-2 are equally sensitive to MMS treatment; Student's t-test: P = 0.3000 and P = 0.2400, respectively. **(B)** SSBs repair kinetics determined by comet assay. Explants of 7d protonemata were treated with 30 mM MMS for 1 h. Repair kinetics of SSBs was measured by comet assay as % of remaining damage after the 0, 3, 5, 10, 20, 60 and 180 min of repair recovery (see legend for Figure 4 in main text). Error bars indicate SD.
